# Robust Sandwich‐Structured Nanofluidic Diodes Modulating Ionic Transport for an Enhanced Electrochromic Performance

**DOI:** 10.1002/advs.201800163

**Published:** 2018-06-26

**Authors:** Qianqian Zhang, Qirong Liu, Jianxin Kang, Qingjiao Huang, Zhaoyue Liu, Xungang Diao, Jin Zhai

**Affiliations:** ^1^ The College of Materials Science and Engineering Beijing University of Technology Beihang University Beijing 100124 P. R. China; ^2^ Key Laboratory of Micro‐Nano Measurement Manipulation and Physics of Ministry of Education School of Physics and Nuclear Energy Engineering Beihang University Beijing 100191 P. R. China; ^3^ Key Laboratory of Bio‐Inspired Smart Interfacial Science and Technology of Ministry of Education Beijing Key Laboratory of Bio‐Inspired Energy Materials and Devices School of Chemistry Beihang University Beijing 100191 P. R. China

**Keywords:** electrochromic performance, ion rectification, nanochannels, nanofluidic diodes, sandwich structures

## Abstract

Biomimetic solid‐state nanofluidic diodes have attracted extensive research interest due to the possible applications in various fields, such as biosensing, energy conversion, and nanofluidic circuits. However, contributions of exterior surface to the transmembrane ionic transport are often ignored, which can be a crucial factor for ion rectification behavior. Herein, a rational design of robust sandwich‐structured nanofluidic diode is shown by creating opposite charges on the exterior surfaces of a nanoporous membrane using inorganic oxides with distinct isoelectric points. Potential‐induced changes in ion concentration within the nanopores lead to a current rectification; the results are subsequently supported by a theoretical simulation. Except for providing surface charges, functional inorganic oxides used in this work are complementary electrochromic materials. Hence, the sandwich‐structured nanofluidic diode is further developed into an electrochromic membrane exhibiting a visual color change in response to redox potentials. The results show that the surface‐charge‐governed ionic transport and the nanoporous structure facilitate the migration of Li^+^ ions, which in turn enhance the electrochromic performance. It is envisioned that this work will create new avenues to design and optimize nanofluidic diodes and electrochromic devices.

## 1 Introduction

Synthetic solid‐state nanochannels that mimic natural ion channel proteins exhibit similar or even superior ionic transport modulations, higher robustness, and stability compared to their bioanalogues. As one of the promising nanofluidic components, they have been used in various fields, like biosensing,^1^ energy conversion,^2^ drug delivery,^3^ and so on. Until now, substantial studies on the construction of nanochannels have focused on the interior surface modifications because unique ionic transport behaviors usually originate from the interactions between ions and surface charges or groups in nanoconfined spaces.^4^ Although the subunits located at the extracellular entrances of the natural channel proteins play the same important function as those at the inner,^5^ less attention has been paid to the functionalization on the exterior surfaces of biomimetic nanochannels and the influence of exterior surfaces on the transmembrane ionic transport. Recently, Li et al. have demonstrated that the introduction of the functional elements at the outer surface of nanochannels contributed to an enhancement of the ion gating behavior owing to a synergistic effect in alliance with the inner‐wall functional elements.^6^ However, related experimental researches concerning ion rectification still remain unexplored as far as we know.

The proposed molecular theory and numerical simulation have predicted that tailoring charges on the exterior surfaces could induce the rectified ionic transport in a nanopore or nanochannel.^7^ As reported, the charged exterior surface has a similar electrostatic influence as charged interior one on the mobile ions at the entrance of nanopore/channel, and thus affect the ion conductance.^8^ For the model of a nanoporous membrane carrying opposite charges on the two exterior surfaces, excess surface charges could accumulate counterions at the pore entrances, leading to a lower access resistance for counterions but a higher one for coions.^9^ In this case, there are significant differences in the transmembrane ion conductance at forward and backward biases, which contributes to an ion rectification behavior analogous to the p–n junction in semiconductor.^10^ Therefore, it is feasible to construct nanofluidic diode by creating opposite charges on the exterior surfaces of a nanopore/channel. Moreover, this strategy would simplify the manufacturing process of nanofluidic diodes compared with classical preparation that requires a fine modification of surface charge in nanoconfined environments.^4^

In this work, we present a rational design of robust nanofluidic diodes based on an anodic aluminum oxide (AAO) nanoporous membrane sandwiched between a tungsten oxide (WO_3_) and a nickel oxide (NiO) thin layer. In the neutral aqueous solution, the membrane is composed of approximately neutral nanopores with two exterior surfaces carrying opposite charges due to different isoelectric point (pI) values of the three components. This broken symmetry in the surface charges generates an ion rectification behavior due to the potential‐induced reorganization of ions within the nanopores, which is further confirmed by the calculated ion concentration distributions at forward and backward biases. The two functional components, WO_3_ and NiO, are chosen elaborately because they are complementary electrochromic materials. Considering a high transparency of the AAO, the sandwich‐structured nanofluidic diode is further developed into an electrochromic membrane. The surface‐charge dominant ionic transport and the nanoporous structure facilitate the migration of Li^+^ ions, which enhance the electrochromic performance including optical modulation and response time. These findings may help to optimize the construction of nanofluidic diode and electrochromic device.

## 2 Results and Discussion

### 2.1 Construction of Sandwich‐Structured Nanofluidic Diodes

**Figure 1:**
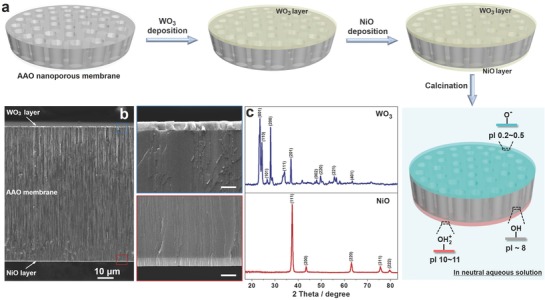
Sandwich‐structured nanofluidic diodes. a) Fabrication flow chart of the sandwich‐structured nanofluidic diodes and the as‐prepared membrane with different charge distribution in neutral aqueous solution stemming from the protonation/deprotonation. b) Cross‐sectional SEM image and the magnified views of the selected areas of AAO nanoporous membrane sandwiched between a WO_3_ and a NiO thin layer. The scale bars in the magnified panels are 1 µm. c) XRD patterns of WO_3_ and NiO layers of the sandwich‐structured membrane display well‐resolved diffraction peaks, indicating a hexagonal WO_3_ and a face‐centered cubic NiO.

The sandwich‐structured nanofluidic diodes were prepared via a facile fabrication process as illustrated in **Figure** **1**a. First, WO_3_ and NiO thin layers were separately deposited on the two surfaces of a commercially available AAO nanoporous membrane through reactive direct‐current (DC) magnetron sputtering.^11^ Next, the sandwich‐structured membrane was annealed at 500 °C under ambient air for 2 h to induce the crystallization of WO_3_ and NiO. The two as‐deposited layers were much thinner compared to AAO membrane (≈83.4 µm thick), and their thicknesses were controlled to be several hundred nanometers by regulating the magnetron sputtering time (Figure 1b). The AAO membrane had cylindrical nanochannels with uniform pore size and high pore density (Figure S1, Supporting Information), and its ionic conductivity was determined to be ≈3.38 µS (Figure S2, Supporting Information). The WO_3_ and NiO thin layers were composed by nanoparticles as shown in Figure S3 (Supporting Information). The intergranular gaps on the sandwich‐structured membrane acted as channels for ions flow. During the magnetron sputtering coating, a layer composed of nanoparticles could form rapidly on the surface of AAO nanoporous membrane under a high power, which thus prevented the formation of metallic oxide nanoparticles in the nanochannels. This was proved by the distributions of nickel and tungsten elements in the marginal areas of the membrane (Figure S4, Supporting Information) and negligible change of the pore size after the deposition of metallic oxide layers (Figure S5, Supporting Information). As shown in Figure 1c, X‐ray diffraction (XRD) pattern of the top layer matched well with the hexagonal WO_3_ (JCPDS card No. 75–2187), and that of the bottom layer was indexed to a face‐centered cubic NiO characterized with (111), (200), (220), (311), and (222) orientations (JCDPS card No. 47–1049). The three inorganic oxides that made up the membrane have distinct pI values.^12^ In a neutral aqueous solution, the surface of AAO nanoporous membrane (pI ≈ 8) is close to an electrical neutrality, while NiO (pI = 10–11) and WO_3_ (pI = 0.2–0.5) carries positive and negative charges respectively, resulting from the protonation and deprotonation of surface hydroxyls.^13^ The two oppositely charged exterior surfaces are essential to realize ion rectification characteristic in this sandwich‐structured nanoporous membrane.[[qv: 7a–c]]

### 2.2 Ion Rectification Property and the Mechanism Based on Theoretical Simulation

**Figure 2:**
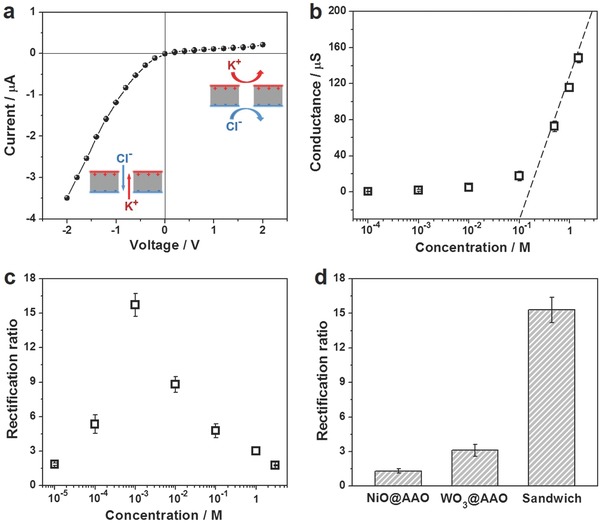
Nanofluidic diode behavior. a) Ionic current–voltage (*I–V*) curve of the sandwich‐structured nanofluidic diodes with a AAO pore size of ≈19.5 nm obtained in 1 × 10^−3^ m KCl electrolyte (pH = 7.2). Insets demonstrate the ionic conduction and nonconduction states. The anode faced NiO side (carrying positive charges) of the membrane. b) The dependence of transmembrane ionic conductance on the concentration of KCl electrolyte (pH = 7.2). The ionic conductance (squares) that deviated from the bulk conductance (dashed line) indicated a surface charge governed ion transport. c) Evolution of ion rectification ratio with the concentration of the electrolyte. d) The ion rectification ratio calculated from the *I–V* curves of sandwich‐structured nanofluidic diodes and AAO nanoporous membrane individually covered by NiO or WO_3_ thin layer measured using the same conditions as panel (a).

The ionic transport behavior of the sandwich‐structured nanofluidic diodes was studied through the current–voltage (*I–V*) measurements (Figure S6, Supporting Information). The anode faced the positively charged surface of the membrane during the test. As shown in **Figure** **2**a, the *I–V* curve exhibits a significant non‐Ohmic behavior indicating a remarkable ion rectification. The positive current was much smaller than the negative current, which suggested that the membrane demonstrated a low ionic conductivity (0.11 µS) at forward bias but a much higher one (1.75 µS) at backward bias (insets of Figure 2a). Here, the rectification ratio, defined as the absolute value of the current ratio at −2 and +2 V, was used to evaluate the rectifying efficiency. For sandwich‐structured nanofluidic diodes with a 20 nm pore size, the ion rectification ratio reached 16.5 in 1 × 10^−3^ m KCl solution (pH 7.2). Figure 2b reveals the dependence of transmembrane ionic conductance on the concentration of the electrolyte. The dashed line represents the ionic conductance of the bulk solution, which is proportional to the electrolyte concentration. However, the transmembrane ionic conductance apparently deviates from the bulk value at below 0.5 m, indicating a surface‐charge dominant ionic transport was applicable to our sandwich‐structured nanofluidic diodes with charged exterior surfaces.^14^ Therefore, the ion rectification behavior could be achieved even in electrolytes of high concentration close to 1 m. The ion rectification behavior of our sandwich‐structured membrane nanofluidic diodes was sensitive to the concentration of the electrolyte. At a low concentration (0.01 × 10^−3^ m), the potential between the two sides of the nanoporous membrane was smaller than the actual transmembrane voltage because of the concentration polarization occurring at the entrances of the nanopores,[[qv: 10a,15]] which resulted in a small rectification ratio of ≈1.8. The increasing concentration weakened the concentration polarization and thus led to an enhancement in the rectification ratio (Figure 2c). After the maximum value at 1 × 10^−3^ m, the ratio decreased gradually with a further increase of the concentration due to a decrease of the electric double layer (EDL) thickness.[[qv: 7a]] This variation trend was in consistent with the bipolar ionic diodes reported previously,[[qv: 10a,16]] which further corroborated the surface‐charge governed ion transport in this system.

When removing WO_3_ or NiO layer from the sandwich‐structured nanofluidic diodes, the ion rectification behavior sharply declined with the rectification ratio dropping to about 1.3 (NiO@AAO) and 3.1 (WO_3_@AAO) shown in Figure 2d and Figure S7 (Supporting Information). This result demonstrated that the sandwich structure with oppositely charged exterior surfaces was key to achieving a strong ion rectification. Except for opposite charges, there are some other factors that could affect the ionic transport, such as steric hindrance,^17^ wettability,^18^ and so on. In the sandwich‐structured membranes, WO_3_ and NiO layers had quite similar steric effect. Hence, coating metallic oxide layers on the AAO membrane made little contribution to the asymmetric ionic transport but decreased the transmembrane ionic conductance (Figure S8, Supporting Information). In view of wettability effect, AAO membrane exhibited hydrophilicity and its surface water contact angle (CA) was determined to be 65° ± 1°. The CA of NiO layer was 51° ± 2° and that of WO_3_ layer was 66° ± 2° (Figure S9, Supporting Information). So, there was no significant wettability difference between the two sides of the membrane after the deposition of metallic oxide layers. The wettability had little influence on the asymmetric transmembrane ionic transport.

**Figure 3:**
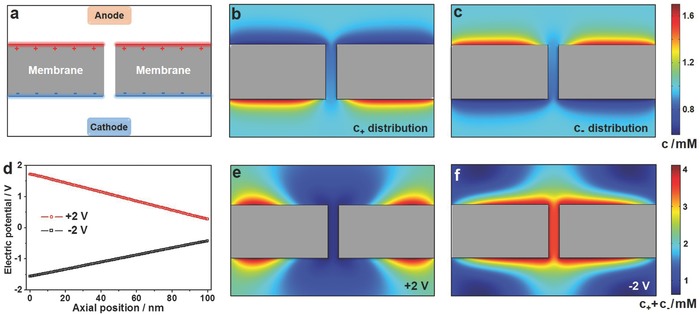
Numerical simulation of the ion rectification. a) The proposed 2D single nanopore membrane model that had a neutral inner wall and two oppositely charged exterior surfaces. b,c) The calculated color maps of b) cation and c) anion distributions in equilibrium (applying no voltage across the membrane). d) Electric potential distributions along the axial direction of the nanopore at forward (+2 V) and reverse (−2 V) biases. The position of *x* = 0 nm corresponded to the positively charged surface of the membrane. e,f) The calculated color maps of ion concentration at e) forward and f) reverse biases. The ion depletion at positive bias and ion enrichment at negative bias contributed to an ion rectification. The electrolyte used in the numerical simulation was 1 × 10^−3^ m KCl. The drawing was not in real scale.

To clarify the ion rectification mechanism, a theoretical simulation was performed based on Poisson–Nernst–Planck (PNP) equations. Here, our sandwich‐structured nanofluidic diode model was simplified to be a single nanopore membrane whose inner surface was electrically neutral and two exterior surfaces had charges of opposite sign but equal density (**Figure** **3**a and Figure S10, Supporting Information). The membrane was mounted between two reservoirs containing 1 × 10^−3^ m KCl electrolyte and the positively charged side faced the anode. More detailed parameters and calculations could be found in Section 4 in the Supporting Information. The potential‐induced reorganization of ionic concentration within the nanopore exhibited distinct behaviors at forward and backward biases, which was the main cause of the ion rectification characteristic. In equilibrium (applying no transmembrane voltage), counterions were adsorbed on the two charged surfaces of the membrane to form EDL shown in Figure 3b,c. Consequently, the charged surfaces would impede the transport of coions but facilitate counterions.^19^ When applied a forward (+2 V) and a reverse (−2 V) bias across the membrane respectively, there formed nonuniform electric potential distributions along the axial direction of the nanopore (Figure 3d), which in turn led to a dramatic change in the ion concentration distribution. Under forward bias, both cations and anions were depleted in the nanopore because of high electrical resistances on the entrances produced by charged exterior surfaces.^9^ This resulted in a fairly low ion concentration in the nanopore at +2 V (Figure 3e) and thus the ionic current was extremely small. Conversely, cations and anions accumulated in the nanopore at a reverse bias owing to low entrance resistances. The remarkable increase of ion concentration inside the nanopore at −2 V created a high overall ionic current (Figure 3f). Meanwhile, the calculated *I–V* curve demonstrated a nonlinear behavior that intuitively reflected the ion rectification behavior of this model (Figure S11, Supporting Information).

**Figure 4:**
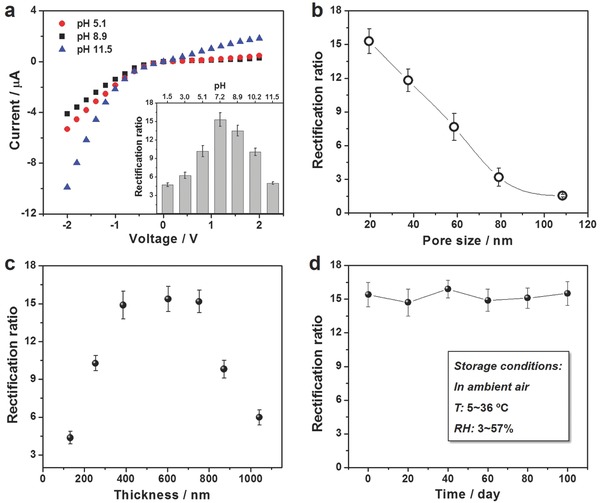
The influence of some factors on the ion rectification. a) *I–V* curves of the sandwich‐structured nanofluidic diodes recorded in 1 × 10^−3^ m KCl with different pH values. Inset shows the ion rectification ratios corresponding to a series of pH values. b, c) Evolutions of ion rectification ratio with b) the average pore size of AAO nanoporous membrane and c) the mean thickness of WO_3_ and NiO layers. d) The ion rectification ratio remained almost a constant in 100 days, indicating a good air stability of the sandwich‐structured nanofluidic diodes. The electrolytes used in all measurements were 1 × 10^−3^ m KCl and the pH values were ≈7.2 in panels (b–d). The average diameter of AAO nanochannels was ≈19.5 nm in panels (a,c,d), and the mean thicknesses of the WO_3_ and NiO layers were ≈386.2 nm in panels (a,b,d).

The sandwich‐structured nanofluidic diodes exhibited a pH‐tunable ion rectification property because the surface charges of three components were sensitive to the pH value of the electrolyte as discussed above.^13^ At low pH values, the exterior surface covered with WO_3_ layer carried a little negative charges due to a weak deprotonation of the surface hydroxyls, while the inner walls of AAO nanopores and the exterior surface covered with NiO were both positively charged owing to the protonation of hydroxyls. With an increase of pH value, the rectification ratio gradually increased and reached ≈11.6 at a pH of 5.1 owing to an enhancement of negative charge density on WO_3_ surface following its increasing deprotonation degree (**Figure** **4**a). At pH = 7.2, the inner walls of AAO nanopores were nearly neutral and the two exterior surfaces had opposite charges with high densities, which contributed to a high rectification ratio of ≈15.4 (inset of Figure 4a). The surface charge density of NiO‐covered surface decreased when the pH value increased to 8.9, leading to a slight decrease of the ion rectification ratio. With a further increase of pH value, the exterior surface covered with NiO converted to be nearly neutral and the rest of the membrane were all negatively charged. In this case, the rectification ratio decreased significantly to be ≈5.4 at a pH of 11.5.

Next, we investigated the dependence of ion rectification behavior on the inherent quality of sandwich‐structured nanofluidic diodes including the pore size of AAO nanoporous membrane and the thickness of WO_3_ and NiO layers. This provided the basis for optimizing the fabrication parameters toward the construction of high‐performance nanofluidic diodes. Figure 4b shows that the rectification ratio decreases with the increasing of pore size. As we discussed above, the distinct actions of surface charges on coions and counterions determined the ion rectification.^19^ Stemming from the constant EDL thickness in the same electrolyte,^20^ the facilitation and obstruction of surface charges to the ion transport was weakening with increasing pore size, which in turn decreased the ion rectification efficiency. The thickness of the charged layer was another influence factor on the ion rectification that could be facilely controlled by modulating the sputtering time‐dependent thickness of WO_3_ and NiO layer. As shown in Figure 4c, the rectification ratio first increased with an increasing thickness because the growing amount of metallic oxides contributed to an enhancement in the surface charge density (Figures S12 and S13, Supporting Information). Then, the rectification ratio reached a higher value of ≈14.9 at 386.2 nm and remained almost stable when the thickness increased to 752.2 nm. This is mainly ascribed to that the charge densities of these charged layers have realized the maximum ability to dominate the ionic transport. Here, we could obtain a desired range of the charged layer thickness for optimizing the fabrication of sandwich‐structured membrane. With a further increase of the thickness, the formation of some large cracks was not conducive to the surface‐charge dominates (Figure S12d, Supporting Information), which in turn led to a dramatic decrease in the rectification ratio (Figure S13, Supporting Information). The thick compact layers are more likely to block the orifices of the AAO nanochannels, which reduces the probability of ions passing the whole membrane. Therefore, the transmembrane ionic current decreased when the AAO membrane was covered with thicker charged layers. (Figures S12d and S13, Supporting Information). Furthermore, the sandwich‐structured nanofluidic diodes exhibited excellent air stability. The ion rectification ratio remained stable in a broad range of temperature and relative humidity (Figure 4d and Figure S14, Supporting Information).

### 2.3 Enhanced Electrochromic Performance Stemming from Controllable Ion Transport

**Figure 5:**
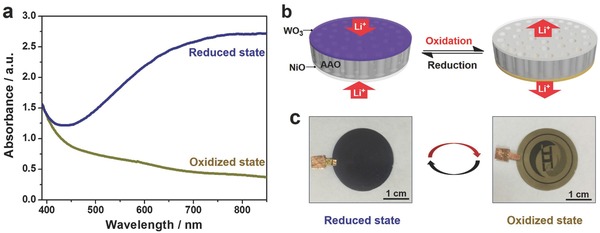
Reversible electrochromic behavior and principle. a) Absorbance spectra of the sandwich‐structured nanofluidic diodes within the wavelength ranging from 400 to 850 nm when applying an oxidation (+1 V vs Ag/AgCl in 3.5 m KCl solution) and a reduction potential (−1.5 V vs Ag/AgCl in 3.5 m KCl solution). b) Schematic drawing for the electrochromic behavior of sandwich‐structured nanofluidic diodes accompanying with intercalation/extraction of Li^+^ ions in/from WO_3_ and NiO layers under reduction and oxidation potentials. c) Optical images of reduced and oxidized membrane on a patterned paper.

In this system, WO_3_ and NiO are well‐known complementary inorganic electrochromic materials whose optical properties could be modulated by the redox potentials.^21^ Considering a high transparency of the AAO membrane (Figure S15, Supporting Information), our sandwich‐structured nanofluidic diodes could be further developed into an electrochromic membrane exhibiting a visual color change. To apply potentials, thin indium tin oxides (ITOs) conducting layers were sputtered on the two sides of AAO nanoporous membrane before the deposition of WO_3_ and NiO. As shown in **Figure** **5**a, there was a decided change in the absorbance of the membrane when switching the applied potential from a reduction one (−1.5 V vs Ag/AgCl in 3.5 m KCl solution) to an oxidation one (+1 V vs Ag/AgCl in 3.5 m KCl solution), especially for the spectrum corresponding to the wavelength over 700 nm. The electrolyte used here was 0.1 m LiClO_4_ contained propylene carbonate (PC) solution. Here, we defined the optical modulation rate (ΔA) as the maximum absorbance difference between the reduced and oxidized states. The ΔA reached 86.1% of the initial value after 100 cycles of alternate application of the redox potentials (Figure S16, Supporting Information), indicating a good reversibility and stability of the optical modulation. The electrochromic behavior of the sandwich‐structured nanofluidic diodes was attributed to the reversible intercalation/extraction of Li^+^ ions in/from the WO_3_ and NiO layers during the redox reaction.^21^ A reduction potential drove electrons into the electrochromic layers accompanying with the insertion of Li^+^ ions (Figure 5b), which resulted in a coloration of WO_3_ but a bleaching of NiO due to the decrease in valences of the metal ions. Consequently, the membrane appeared in dark blue that is a color of the reduced WO_3_ (Figure 5c). Conversely, the colored WO_3_ was bleached, while NiO was the opposite with the extraction of Li^+^ ions driven by the oxidation potential, which caused the color of the membrane to turn tawny. It is worth mentioning that the annealing temperature was reduced to 300 °C because the compact films prepared at a higher temperature were too dense for ion insertion during the reduction process.^22^ In this case, the sandwich‐structured nanofluidic diodes exhibit an ion rectification behavior with a lower ratio of 8.2 compared with those annealed at 500 °C (Figure S17, Supporting Information).

**Figure 6:**
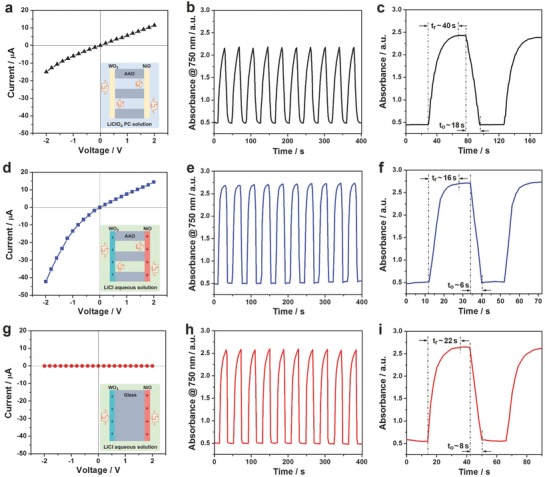
Sandwich‐structured nanofluidic diodes modulating ionic transport for an enhanced electrochromic behavior. a,d,g) *I–V* behaviors of sandwich‐structured nanofluidic diodes recorded in 0.1 m LiClO_4_ PC solution a), in d) 0.1 m LiCl aqueous solution and g) sandwich‐structured glass membrane measured in 0.1 m LiCl aqueous solution. b,e,h) Corresponding in situ absorbance variations at a wavelength of 750 nm in response to periodic redox potentials of +1 and −1.5 V for 20 s per step. c,f,i) In situ absorbance evolutions with extended time under alternating redox potentials for the comparison of electrochromic response time measured at different conditions.

As discussed above, the sandwich‐structured nanofluidic diodes modulated ionic transport through the membrane, which could influence the electrochromic performance that depends on the cation migration between the electrochromic layers and the electrolyte.^21^ To evaluate the effect of ionic transport on the electrochromic performance, several systems were established on the basis of different electrolyte properties (aqueous or organic solution) and membrane structures (nanoporous or nonporous membrane). As shown in **Figure** **6**a, a linear *I–V* property indicated that the ion rectification behavior of the sandwich‐structured nanoporous membrane could not be obtained in the LiClO_4_ PC electrolyte, owing to no charge generation on the surface of the membrane in an organic solution.^13^ In this case, the membrane demonstrated a reversible change in the absorbance (ΔA = 1.68) at the wavelength of 750 nm in response to the alternately applied redox potentials of +1 and −1.5 V (Figure 6b). The response time was another important parameter to evaluate the electrochromic performance.[[qv: 21b]] As seen from Figure 6c, the response time was ≈40 s for the reduction process (*t*_r_) and 18 s for the oxidation process (*t*_o_). A longer *t*_r_ may be ascribed to that the crystallization of the electrochromic layers made it difficult for Li^+^ ions intercalation.^22^

In order to understand how ionic transport modulation of the sandwich‐structured nanofluidic diodes affects the electrochromic performance, the aforementioned organic electrolyte was replaced by a LiCl aqueous solution (pH 6.8) with the same concentration. In this situation, the sandwich‐structured nanoporous membrane carried opposite charges on the two exterior surfaces, which could regulate ionic transport reflected from the ion rectification behavior (Figure 6d).[[qv: 7a–c]] A transmembrane ionic current was higher than that obtained in organic solution especially for the reverse current. This was ascribed to the combination of the surface charge‐governed ionic transport, the enhanced ionic conductivity, and the introduction of extra mobile ions. Consequently, a larger optical modulation per unit time (ΔA = 2.20) was achieved as shown in Figure 6e. Moreover, the charged exterior surfaces of the sandwich‐structured nanoporous membrane facilitated the intercalation of Li^+^ ions in WO_3_ layer and the extraction of them out from NiO layer,^19^ which contributed to a faster response time, particularly that for the reduction process (*t*_r_ = 16 s, Figure 6f). Finally, we studied the influence of nanochannels on the electrochromic performance using a piece of glass as an alternative to the AAO membrane. As shown in Figure 6g, ions could not pass through the membrane in a LiCl aqueous solution. This made Li^+^ ions flow in and out of the electrochromic layers from one side rather than from two sides when employing a nanoporous membrane (Figure 6d). A decrease in the entrance and exit areas of Li^+^ ions led to a slight degradation of the electrochromic performance with a smaller optical modulation and a longer response time (Figure 6h,i). These results indicated that the ionic transport modulation of the sandwich‐structured nanofluidic diodes could improve the electrochromic performance.

## 3 Conclusion

In summary, we presented for the first time the rational fabrication of robust sandwich‐structured nanofluidic diodes. The experimental evidences demonstrated that the highly efficient control over the ionic transport through the nanoporous membrane could be achieved by modifying opposite charges on the exterior surfaces using two inorganic oxides with distinct pI values, which was supported by the calculated ion concentration distributions in our theory model. By utilizing complementary electrochromic materials as the functional components, the sandwich‐structured nanofluidic diodes were further developed into an electrochromic membrane whose optical modulation and response time could be enhanced by modulating ionic transport. We expect this work can provide a new insight into the design and optimization of nanofluidic diode and electrochromic device.

## 4 Experimental Section

*Sandwich‐Structured Nanofluidic Diode Preparation*: WO_3_ and NiO films were separately deposited onto the two sides of commercially available AAO nanoporous membranes (*Φ* = 2.5 cm, Puyuan Nano, China) via reactive DC magnetron sputtering using a magnetron sputtering coating system equipped with pure tungsten and nickel target.^11^ The average pore density of AAO membrane with 20 nm pore diameter is ≈3.3 × 10^10^ cm^−2^. Deposition of WO*_x_* film was performed in a mixture of O_2_ (99.99%) and Ar (99.99%) with a mass flow ratio of 33%, during which the sputtering pressure and power were kept at 2.0 Pa and 320 W. The sputtering parameters of NiO*_x_* were O_2_/Ar mass flow ratio of 12.8%, a sputtering pressure of 1.5 Pa and a working power of 210 W. The two metallic oxides were determined to be WO_2.47_ and NiO_0.87_ through the X‐ray photoelectron spectroscopy. Subsequently, as‐prepared membranes were annealed at 500 °C under ambient air for 2 h to induce WO_3_ and NiO crystallization.

*Characterization*: The morphologies of sandwich‐structured nanofluidic diodes were observed by a Quanta FEG 250 environmental scanning electron microscope (SEM) (FEI, USA). A Shimadzu XRD‐6000 X‐ray diffractometer using Cu Kα radiation (40 kV, 40 mA) was used to investigate the crystal structures of WO_3_ and NiO.

*Ion Rectification Study*: A sandwich‐structured membrane was mounted between two chambers of a homemade electrochemical cell filled with the electrolyte of KCl (Beijing Chemical Factory, China) solution (Figure S6, Supporting Information). The pH value of the electrolyte was adjusted using 1 mol L^−1^ HCl and KOH (Beijing Chemical Factory, China) solutions. The anode was fixed to face the WO_3_‐covered surface of the membrane. *I–V* properties were measured by a Keithley 6487 picoammeter (Keithley Instruments, USA) with two Ag/AgCl electrodes applying transmembrane voltages between −2 and +2 V.

*Numerical Simulation*: A theoretical simulation based on PNP equations was performed to support the experimental results on ion rectification in the system (Section 4 in the Supporting Information). To facilitate the calculation, the theoretical model was simplified as a cylindrical nanopore membrane whose inner surface was electrically neutral and two exterior surfaces had charges of opposite sign but equal density (Figure S10, Supporting Information). The pore diameter and channel length was set as 20 and 100 nm, respectively. The anode was set to face the positively charged surface of the membrane. The calculation was performed using the “electrostatics” and “transport of diluted species” modules of the commercial finite element software COMSOL Multiphysics 5.2.

*Electrochromic Performance Study: In situ* optical property evolutions of the sandwich‐structured nanofluidic diodes in response to different redox potentials were studied through the combined use of a UV–vis spectrophotometer (V‐1800 PC, Shanghai Mapada Instruments Co., Ltd., China) and an electrochemical workstation (CHI660E, Shanghai Chenhua Apparatus Co., Ltd., China). During the measurements, the potentials were applied to the membrane having ITO conductive films in a three‐electrode system with a platinum foil serving as counter electrode and Ag/AgCl electrode (in 3.5 m KCl) as a reference. The electrolyte used here were 0.1 m LiClO_4_ contained PC solution and 0.1 m LiCl aqueous solution.

## Conflict of Interest

The authors declare no conflict of interest.

## Supporting information

Supplementary
