# Supplementary material for: Robust Sandwich‐Structured Nanofluidic Diodes Modulating Ionic Transport for an Enhanced Electrochromic Performance
Source: Adv Sci (Weinh). 2018 Jun 26;5(9):1800163. doi: 10.1002/advs.201800163 (PMC6145424; doi:10.1002/advs.201800163)
Supplement: Supplementary file 1 — Supplementary [file ADVS-5-1800163-s001.pdf]

Copyright WILEY-VCH Verlag GmbH & Co. KGaA, 69469 Weinheim, Germany, 2018.

ADVANCED SCIENCE
Open Access

Supporting Information

for *Adv. Sci.,* DOI: 10.1002/advs.201800163

Robust Sandwich-Structured Nanofluidic Diodes Modulating Ionic Transport for an Enhanced Electrochromic Performance

Qianqian Zhang, Qirong Liu, Jianxin Kang, Qingjiao Huang, Zhaoyue Liu, Xungang Diao, and Jin Zhai**

Copyright WILEY-VCH Verlag GmbH & Co. KGaA, 69469 Weinheim, Germany, 2016.

Supporting Information

Robust Sandwich-Structured Nanofluidic Diodes Modulating Ionic Transport for an Enhanced Electrochromic Performance

Qianqian Zhang, Qirong Liu, Jianxin Kang, Qingjiao Huang, Zhaoyue Liu, Xungang Diao, Jin Zhai**

Content of Supporting Information

1. The morphology of AAO nanoporous membrane
2. Ionic conductivity of AAO membrane before and after each layer deposition
3. Surface morphologies of WO_3 and NiO thin layers
4. WO_3 and NiO distributions in the nanochannels
5. *I-V* property measurement setup
6. *I-V* behaviors of WO_3@AAO and NiO@AAO membranes
7. The influence of steric hindrance and wettability on the asymmetric ionic transport
8. Theoretical simulation
9. The effect of charged layer thickness on surface morphology and *I-V* property
10. Distributions of *T* and relative humidity (*RH*) in the period of air-stability study
11. Optical property of AAO nanoporous membrane
12. Cycle ability of the electrochromic performance
13. *I-V* behaviors of sandwich-structured nanofluidic diodes annealed at a low temperature
14. References

1. The morphology of AAO nanoporous membrane

As shown in Figure S1, the same pore size on the two sides and the well-aligned nanotubular arrays of the cross-section both indicated that the AAO nanoporous membrane used here had cylindrical channels. Furthermore, a high pore density provided a high background current of several microamperes.

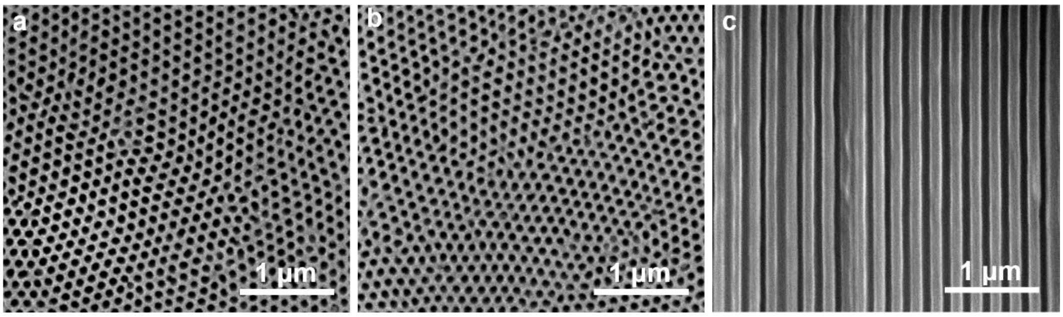

Figure S1. The top views of two sides (a, b) and the magnified cross-sectional image (c) of the AAO nanoporous membrane.

2. Ionic conductivity of AAO membrane before and after each layer deposition

From the *I-V* behaviors (Figure S2a), we can see that the magnitude of transmembrane ionic current decreases when the AAO membrane is covered by a WO_3 layer because of an enhanced steric hindrance. In this case, the ionic conductance declines from 3.43 to 2.48 μS at -2 V, and from 3.33 to 0.57 μS at +2 V (Figure S2b). Then, there is a further reduction in the ionic current after the deposition of a NiO layer on the other side of the AAO membrane. The ionic conductance further drops to 1.75 μS and 0.11 μS at -2 V and +2 V, respectively.

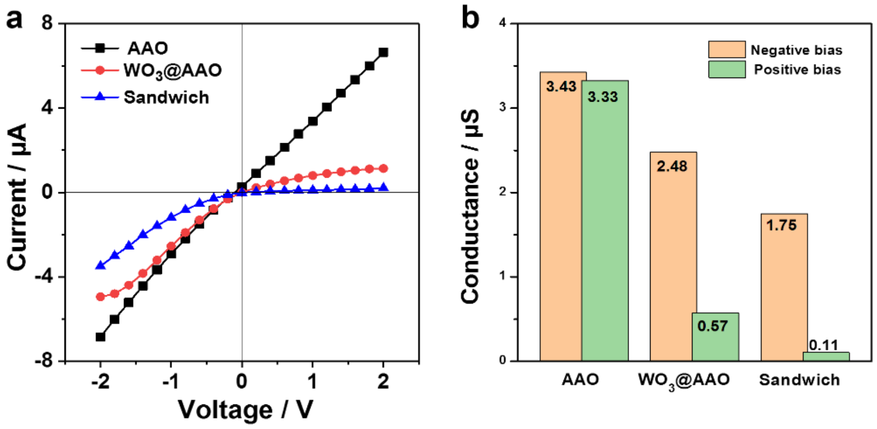

Figure S2. (a) *I-V* behaviors of AAO membrane, WO_3-covered AAO membrane and sandwich-structured membrane obtained in 1 mM KCl solution (pH 7.2). (b) The calculated ionic conductance under negative (-2 V) and positive (+2 V) bias.

3. Surface morphologies of WO_3 and NiO thin layers

The WO_3 and NiO thin layers on the AAO membrane obtained using magnetron sputtering were composed by nanoparticles as shown in Figure S3. The ions would pass the layers through the tiny gaps between the particles.

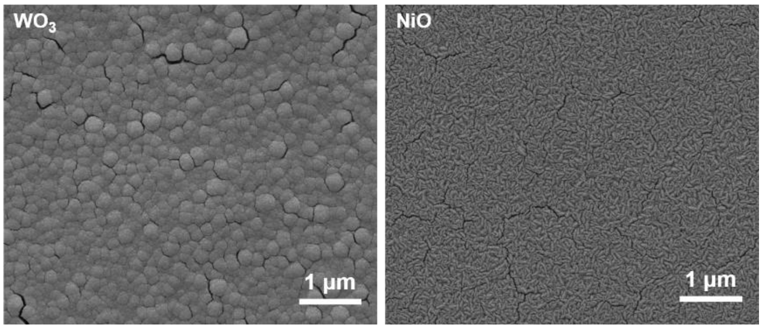

Figure S3. Top-view SEM images of WO_3 and NiO thin layers on the two sides of AAO nanoporous membrane.

4. WO_3 and NiO distributions in the nanochannels

The element distribution mappings in the cross-sectional region of the AAO nanoporous membrane was measured to understand whether the inner surface of the nanochannels was stained by the metallic oxides. As shown in Figure S4, nickel and tungsten elements substantially exist in the two marginal areas of the membrane, and little could be detected in the nanochannel section. Therefore, it is reasonable to believe that there is little WO_3 and NiO deposited in the nanochannels.

Furthermore, the average pore diameter of original AAO membrane is determined to be 19.2 nm, and that is 18.6 nm after the deposition of WO_3 and NiO layers (Figure S5). This negligible variation in the pore size further proves that the interior surface of nanochannels is almost not stained by WO_3 or NiO.

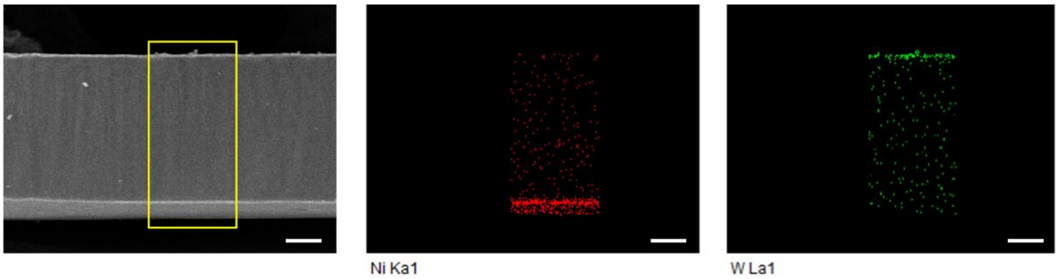

Figure S4. SEM image of the whole cross-section of AAO nanoporous membrane sandwiched between the WO_3 and NiO layers, and element distribution mappings of nickel and tungsten in the selected area (within yellow wireframe). The scale bar is 20 μm.

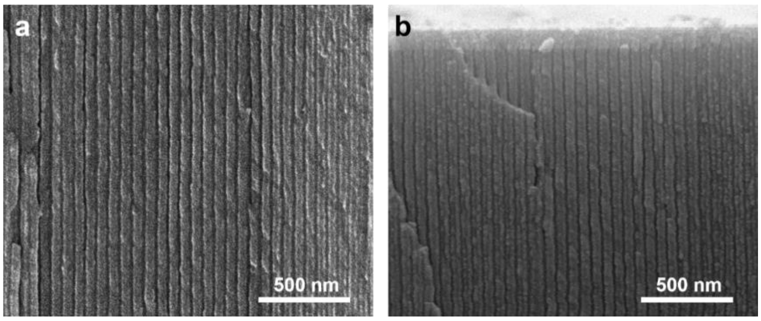

Figure S5. Cross-sectional SEM images of the AAO nanoporous membrane before (a) and after (b) the deposition of WO_3 and NiO layers on the two sides.

5. *I-V* property measurement setup

Figure S6 showes the measurement setup for investigating the *I-V* properties of the sandwich-structured nanofluidic diodes.

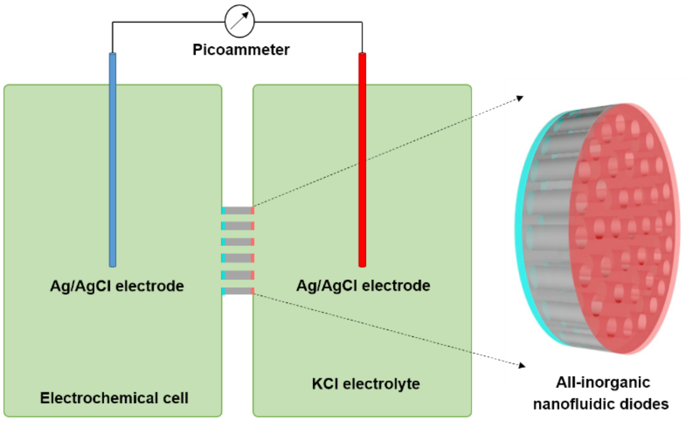

Figure S6. Schematic drawing of the setup for measuring the *I-V* curves.

6. *I-V* behaviors of WO_3@AAO and NiO@AAO membranes

For comparison, the *I-V* properties were measured after individually removed the WO_3 layer and NiO layer from the sandwich-structured nanofluidic diodes in 1 mM KCl electrolyte (pH 7.2). As shown in Figure S7, the AAO nanoporous membrane covered with WO_3 layer exhibited a slight ion rectification with a ratio of 3.1. This could be ascribed to the formation of p-n junction ionic channels in heterogeneous WO_3/AAO nanoporous membrane.[1] However, the ion rectification ratio decreased significantly in an AAO nanoporous membrane covered with NiO layer because the two components both had positive charges with similar density owing to their close pI values.

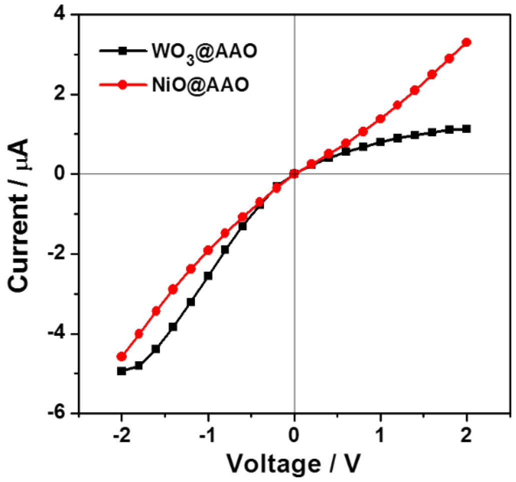

Figure S7. *I-V* behaviors of WO_3@AAO and NiO@AAO membranes.

7. The influence of steric hindrance and wettability on the asymmetric ionic transport

As shown in Figure S8, the magnitude of the ionic current reduces significantly after the AAO nanoporous membrane is sandwiched by the two WO_3 or NiO layers, indicating an effect of steric hindrance on the ionic transport. The WO_3 and NiO layers demonstrate quite similar steric effect because of their overlapping *I*-*V* curves. Note that the AAO membrane exhibits a linear *I*-*V* behavior after the coverage of WO_3 or NiO layers on both sides, while an ion rectification behavior appears when it is sandwiched by a WO_3 and a NiO layer. This suggests that the opposite charge on the outer surface of the membrane is a decisive cause of the asymmetric transmembrane ion transmission. Therefore, the steric hindrance of WO_3 and NiO layer could decrease the transmembrane ionic conductance but has little effect on the asymmetric ionic transport.

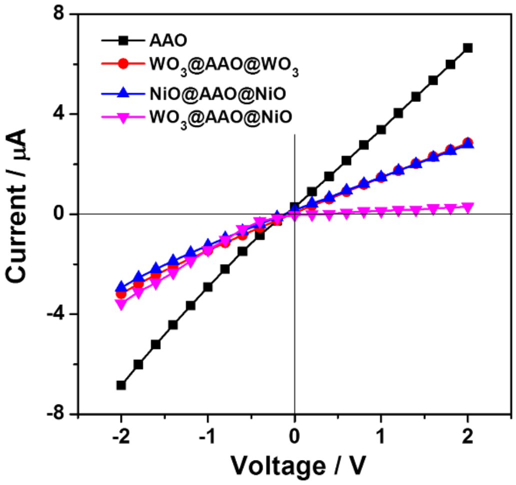

Figure S8. *I*-*V* behaviors of AAO nanoporous membrane, AAO nanoporous membrane sandwiched by the two WO_3 layers, two NiO layers, a WO_3 and a NiO layers measured in 1 mM KCl aqueous solution (pH 7.2). The thickness of the metallic oxide layers were ~400 nm.

In view of wettability effect, original AAO membrane exhibits hydrophilicity and its surface water contact angle (CA) is determined to be 65° ± 1°. After the deposition of two metallic oxide layers, the CA of NiO-covered side is 51° ± 2° and that of WO_3 side is 66° ± 2° (Figure S9). There is no significant wettability difference between the two sides of the

membrane. Furthermore, the sandwich-structured membrane allow the ionic current to flow preferentially from the WO_3 side, which is in conflict with the effect of wettability on fluids. Therefore, the wettability has little influence on the transmembrane ionic transport.

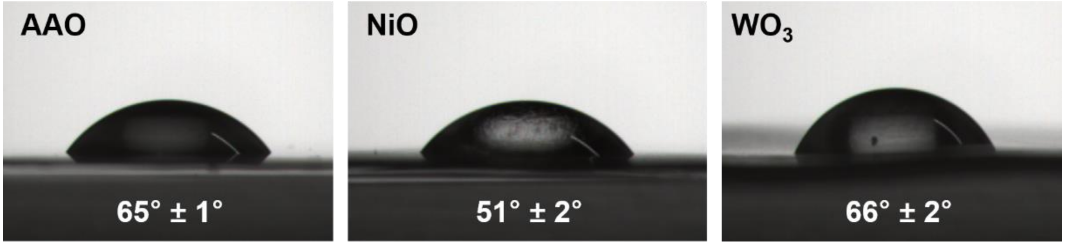

Figure S9. Surface water contact angles of AAO membrane, NiO and WO_3-covered side of sandwich-structured membrane.

8. Theoretical simulation

A theoretical simulation was performed to investigate the ion rectification mechanism of our sandwich-structured nanofluidic diodes. The theoretical model was simplified as a 2D cylindrical nanopore whose inner surface was electrically neutral and the two exterior surfaces carried opposite charges (Figure S10). The pore diameter and channel length was set as 20 nm and 100 nm.

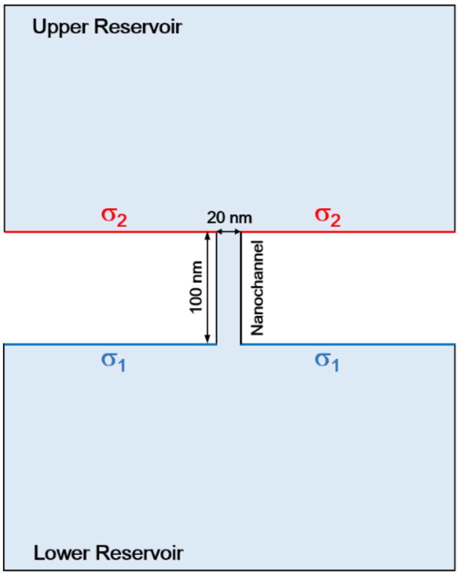

Figure S10. The 2D cylindrical nanochannel model with an electrically neutral inner wall and two oppositely charged exterior surfaces. The figure is not drawn in real scale.

The ion transport in this model was studied by employing the Poisson equation that is related to electrical potential caused by ion concentration (Equation S1), Nernst–Planck (PNP) equation referring to particle flux of ions (Equation S2), coupled with the steady state continuous equation (Equation S3).[2]

$$\nabla^2\varphi = -\frac{F^2}{\varepsilon}\sum z_i c_i \qquad (1)$$

$$J_i = -D_i(\nabla c_i + \frac{z_i F c_i}{RT}\nabla\varphi) \qquad (2)$$

$$\nabla J_i = 0 \qquad (3)$$

Where φ and ε are the electrical potential and the dielectric constant of medium. z_i, c_i, J_i and D_i represent the charge number, concentration, particle flux and the diffusion coefficient of species i, respectively. The electrolyte used here was 1 mM KCl aqueous solution. Therefore, the diffusion coefficient of K^+ and Cl^-, D_{K^+} and D_{Cl^-}, were determined to be 1.96×10^{-9} and 2.03×10^{-9} m^2/s, and the dielectric constant ε was 80.[2a,3] The boundary conditions for the electrical potential and ion flux are,

$$\vec{n}\cdot\nabla\varphi = -\frac{\sigma}{\varepsilon} \quad (4)$$

$$\vec{n}\cdot J_i = 0 \quad (5)$$

Where $\vec{n}$ and σ denote the unit normal vector and surface charge density. In our model, the charge density on the inner wall of the nanochannel was set to zero, and the surface charge density σ_1 and σ_2 were set as -5×10^{-4} and 5×10^{-4} C/m^2, respectively. The bottom edge of the lower reservoir was grounding. The ionic current of species i could be calculated through integrating its particle flux along the cross section of the nanochannel shown in Equation S6, from which the relationship between the ion current and the electrical potential was established.

$$I_i = \int_s J_i ds = -\int_s D_i(\nabla c_i + \frac{z_i F c_i}{RT}\nabla\varphi)ds \quad (6)$$

The following *I-V* behavior obtained through above theoretical calculation shew a good agreement with our experimental demonstrations (Figure S11).

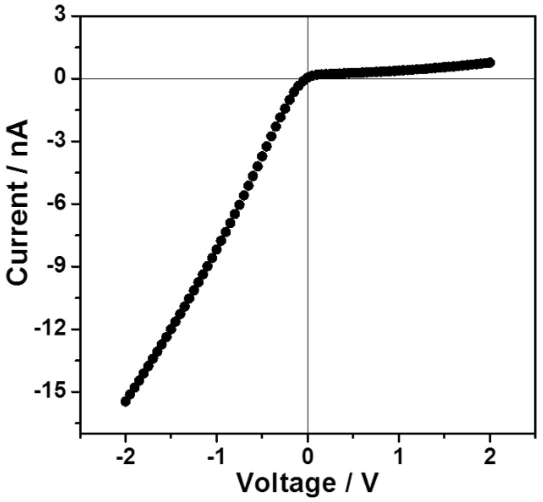

Figure S11. The calculated *I-V* curve based on above theoretical model.

9. The effect of charged layer thickness on surface morphology and *I-V* property

Taking WO_3 as an example in our system, the depositing amount increased with increasing magnetron sputtering time, which directly influenced its coverage on the surface of AAO membrane and its thickness. As shown in Figure S12, the surface coverage increased with the thickness of the WO_3 layer, leading to an enhancement in the rectification ratio (Figure S13). With a further increase of the thickness, the formation of large cracks was not was not conducive to the surface-charge dominates (Figure S12d). Moreover, the compact layer composed of large particles definitely blocked the entrances of ion transport through nanochannels. As a result, there was a decrease in ion rectification ratio and ion current (Figure S13).

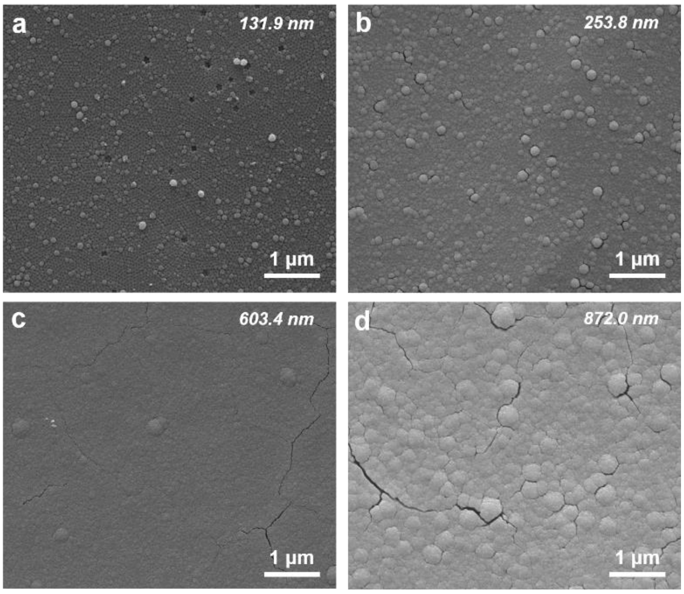

Figure S12. The top-view SEM images for the WO_3 layers corresponding to the thickness of 131.9 nm (a), 253.8 nm (b), 603.4 nm (c) and 872.0 nm (d).

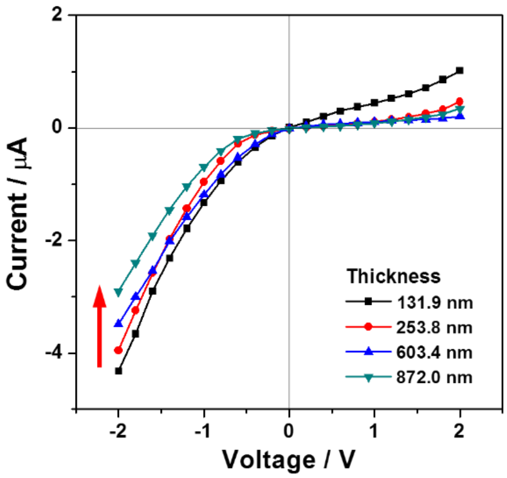

Figure S13. *I-V* properties of sandwich-structured nanofluidic diodes having the charged layer with different thickness.

10. Distributions of *T* and *RH* in the period of air-stability study

During the period of air-stability study, the average highest and lowest temperature ranged from 16 to 34 ºC and from 6 to 25 ºC, respectively (Figure S14a). The relative humidity demonstrated a wide distribution from 3% to 57% shown in Figure S14b.

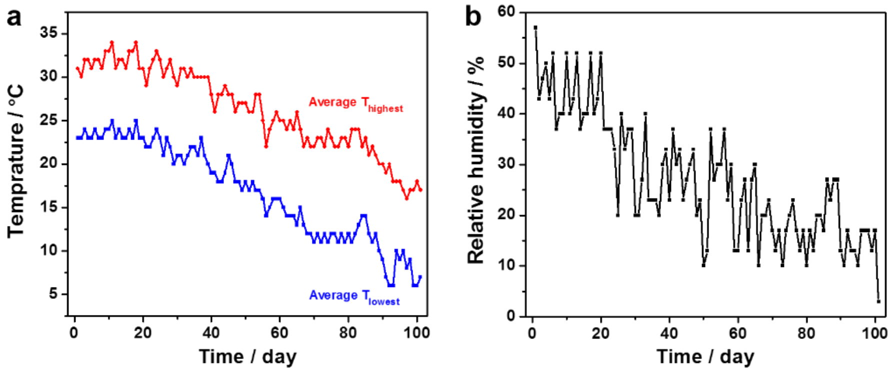

Figure S14. Distributions of the average highest and lowest temperature (a) and relative humidity (b) in the period of air-stability study.

11. Optical property of AAO nanoporous membrane

AAO nanoporous membrane had a high transparency with a transmittance of ~70% and an absorbance of ~0.15 within the wavelength ranging from 400 to 850 nm (Figure S15).

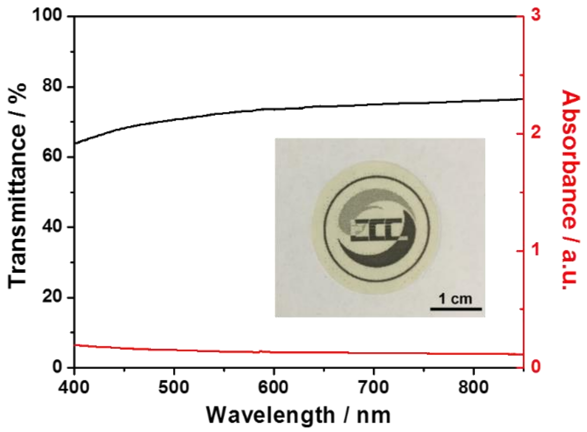

Figure S15. The transmittance and absorbance spectra of AAO nanoporous membrane within the wavelength ranging from 400 to 850 nm. Inset: Photograph of AAO nanoporous membrane on a patterned paper.

12. Cycle ability of the electrochromic performance

The absorbance modulation rate at a wavelength of 750 nm reaches 86.1 % of the initial value after 100 cycles of alternate application of the redox potentials (Figure S16). The limitation of the cycle stability mainly lies in that it is impossible to extract all ions inserted in the electrochromic layers by a constant oxidation potential.

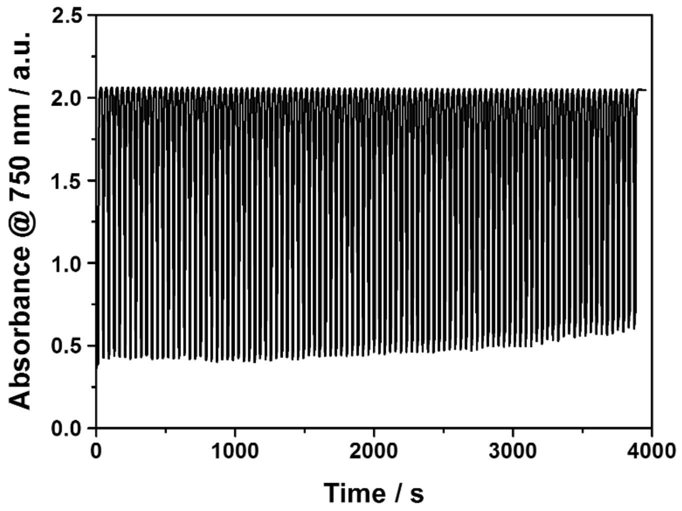

Figure S16. Evolution of absorbance at a wavelength of 750 nm in response to the alternate application of the redox potentials.

13. *I-V* behaviors of nanofluidic diodes annealed at a low temperature

When reduced the annealing temperature to 300 °C, the sandwich-structured nanofluidic diodes exhibit an ion rectification behavior with a ratio of 8.2 (Figure S17). The rectification trend is the same as the nanofluidic diodes obtained at 500 °C, but the rectification ratio is lower. The metallic oxide with a specific crystal has a definable isoelectric point that could be used to determine the surface charge polarity. Reducing the annealing temperature decreases the crystallinity of WO_3 and NiO, which in turn decreases the charge density of the two layers. Therefore, the ion rectification ratio reduces with the reduction of the annealing temperature.

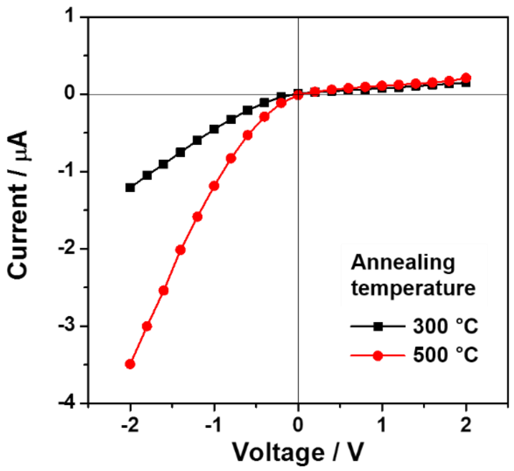

Figure S17. *I-V* curves of sandwich-structured nanofluidic diodes obtained at the annealing temperature of 300 and 500 °C.

14. References

[1] R. Yan, W. Liang, R. Fan, P. Yang, *Nano Lett.* **2009**, *9*, 3820.

[2] a) H. Daiguji, Y. Oka, K. Shirono, *Nano Lett.* **2005**, *5*, 2274; b) I. Vlassiouk, S. Smirnov, Z. S. Siwy, *ACS Nano* **2008**, *2*, 1589.

[3] H. Daiguji, P. Yang, A. Majumda, *Nano Lett.* **2004**, *4*, 137.
